# Dynamic loss of hormone receptors and reshaped drug sensitivity in male breast cancer organoids: A first case report and clinical implications

**DOI:** 10.1515/jtim-2026-0032

**Published:** 2026-04-04

**Authors:** Qing Zhao, Yunting Li, Xiaotong Han, Jingru Wang, Xiaotong Dong, Zhesheng Chen, Dawei Yuan, Yunxiang Zhang

**Affiliations:** Department of Molecular Pathology, Qingdao Central Hospital, University of Health and Rehabilitation Sciences, Qingdao, Shandong Province, China; Center of Precision Pathologic Diagnosis, Weifang People's Hospital, Shandong Second Medical University, Weifang, Shandong Province, China; Department of Basic Medicine, Shandong Second Medical University, Weifang, Shandong Province, China; Department of Pharmaceutical Sciences, College of Pharmacy and Health Sciences, St. John's University, Queens, New York, USA; Geneis (Beijing) Co. Ltd., Beijing, China; School of Basic Medical Sciences, Shandong University, Jinan, Shandong Province, China

## To the editor

Male breast cancer (MBC) is rare (< 1% of all breast cancers) and biologically heterogeneous. Compared with female breast cancer (FBC), it more often presents as hormone receptor–positive (HR+), whereas triple-negative breast cancer (TNBC) is uncommon.^[1]^ Distinct germline and somatic features—*e.g*., a higher prevalence of BRCA2 alterations—limit extrapolation from FBC; although androgen receptor (AR) expression is common in MBC, its prognostic value remains uncertain across studies.^[2]^ Patient-derived organoids (PDOs) enable high-fidelity modeling and individualized drug testing, yet longitudinal HR dynamics in male models are rarely documented. Here, we report a MBC patient-derived organoid (PDO) that shifted from Luminal A to a TNBC-like phenotype during passaging, integrated whole-genome sequencing (WGS) of the primary tumor, adjacent normal tissue, and passage-6 organoids (PD6), and presented exploratory drug-sensitivity data, including zinc pyrithione (ZnPT).

**Figure 1:**
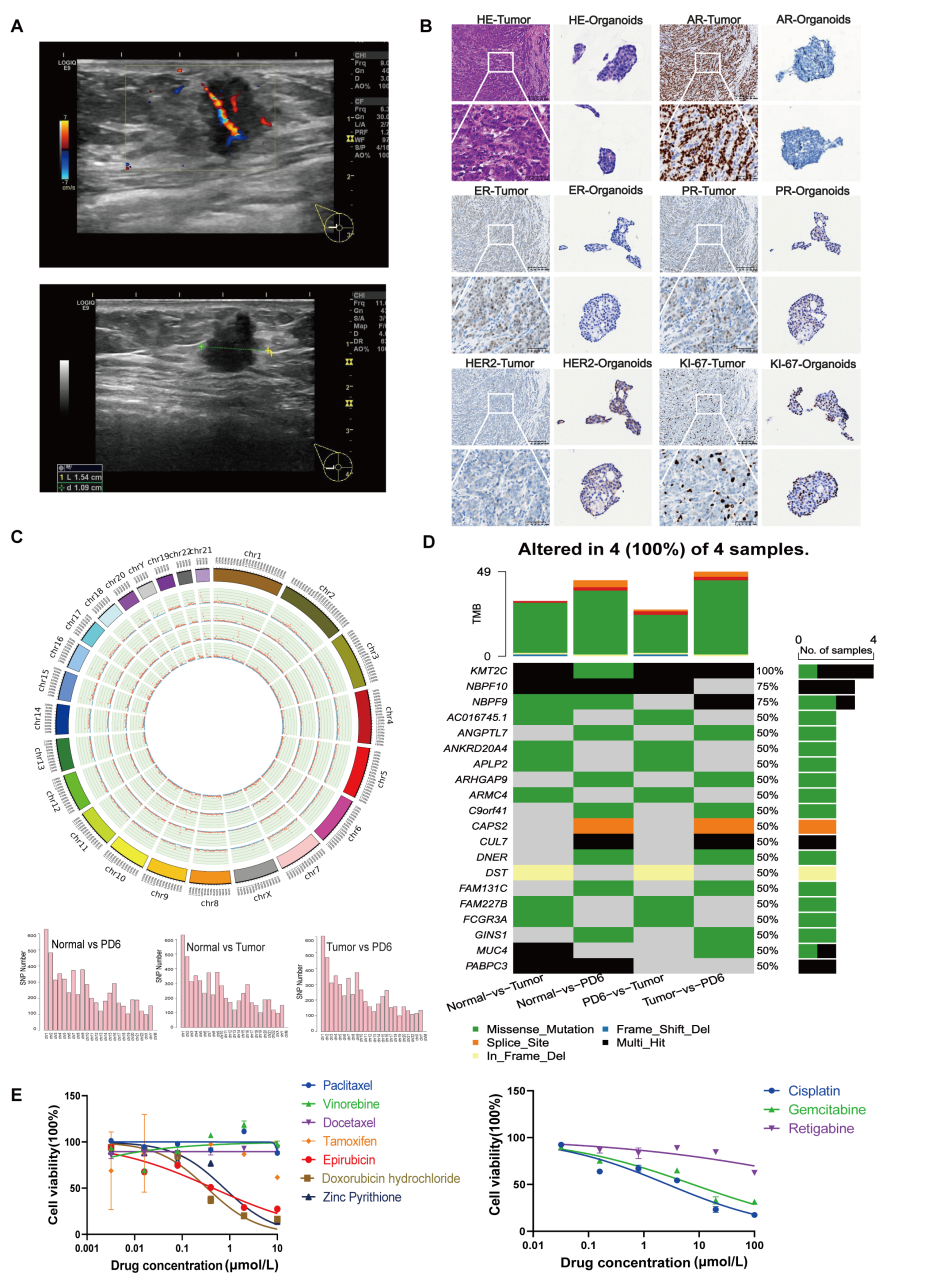
Clinical, genomic, and pharmacologic overview of an MBC PDO. A. Pre-operative ultrasound (BI-RADS 4C). B. Tumor and organoid H & E/IHC: organoids lack ER/PR/AR; HER2 and Ki-67 as indicated (scale: 200 μm; insets: 50 μm). C. 1-Mb SNV/indel density maps (tumor *vs*. PD6). D. Variant-class composition across four pairwise comparisons is summarized, with tumor mutational burden (TMB) shown above. E. Dose-response: relative taxane resistance; lower IC50 for epirubicin/doxorubicin; exploratory ZnPT activity (inset). BI-RADS: Breast Imaging Reporting and Data System; ER: estrogen receptor; PR: progesterone receptor; AR: androgen receptor; IHC: immunohistochemistry; PD6: passage-6 organoids; IC50: inhibitory concentration; ZnPT: zinc pyrithione.

In January 2024, a 77-year-old man presented with a painless right-breast mass (1.8 cm × 1.5 cm × 1.5 cm). Ultrasonography and magnetic resonance imaging (MRI) showed an irregular hypoechoic lesion categorized as Breast Imaging Reporting and Data System (BI-RADS) 4C with enlarged right axillary lymph nodes (0.9 cm × 0.6 cm) (Figure 1A). Core needle biopsy confirmed invasive ductal carcinoma, histologic grade II (score 6: tubule formation 3, nuclear grade 2, mitotic count 1) with vascular and neural invasion. Immunohistochemistry (IHC) on the surgical specimen demonstrated Luminal A features—estrogen receptor (ER) /progesterone receptor (PR)/AR positive; human epidermal growth factor receptor 2 (HER2) negative; Ki-67 index 15%. Tumor tissue was embedded in Matrigel and expanded in growth–factor–supplemented medium to generate PDOs that proliferated for nine months, forming compact spheroids with sharp borders and prominent nucleoli. From passage-4 organoids (PD4) onward, IHC consistently showed loss of ER, PR, and AR; earlier passages were not evaluable because of limited material (Supplement 1). By PD6, receptor negativity was complete, whereas HER2 and Ki-67 remained concordant with the primary tumor—indicating a Luminal A→TNBC-like transition (Figure 1B).

WGS of the primary tumor, adjacent normal tissue, and PD6 identified somatic single-nucleotide variants (SNVs), insertions/deletions (indels), copy-number variations (CNVs), and structural variants (SVs). Chromosome-wide SNV/indel distributions were visualized as 1-Mb density maps (Figure 1C). Variant-class composition (missense, frameshift, stop-gain, splice-site, *etc*.) is summarized separately (Figure 1D), with missense substitutions most prevalent. Relative to normal, the tumor showed alterations (*e.g*., AC016745.1, ANKRD20A4, APLP2, ARMC4, DST, FAM227B, FCGR3A, MUC4); at MUC4, tumor multi-hit and PD6 missense were both absent in normal. PD6 acquired additional alterations over the tumor (*e.g*., KMT2C, NBPF9, ANGPTL7, ARHGAP9, C9orf41, CAPS2, CUL7, DNER, FAM131C, GINS1), consistent with clonal selection and culture-associated drift, while a subset (*e.g*., PABPC3) was shared by tumor and PD6 versus normal. Pathway-level annotation implicated estrogen-response (late), cholesterol homeostasis/sterol regulatory element–binding protein (SREBP) programs, and G2–M checkpoint control (Supplement 2). Although correlative, prior functional data suggest KMT2C loss can uncouple ERα-dependent transcription from estrogen abundance, providing a testable hypothesis for epigenetic reprogramming during culture.

Drug testing evaluated ten FDA-approved chemotherapies plus a small-molecule KCNQ4 agonist using six fivefold dilutions (10.0 μmol/L–3.2 mmol/L or 100 μmol/L–32 mmol/ L) (Table 1) and 4-day ATP-based viability assays; drug effects were summarized by the half-maximal inhibitory concentration (IC50). PD6 organoids were relatively resistant to paclitaxel, vinorelbine, and docetaxel, with lower IC50s for epirubicin (about 0.446 μmol/L), doxorubicin (about 0.337 μmol/L), and cisplatin (about 2.708 μmol/L). Notably, ZnPT showed low-micromolar activity (about 0.767 μmol/L) (Figure 1E). These single-PDO data are descriptive, not therapeutic guidance; ZnPT is not guideline-recommended for TNBC. Importantly, taxanes—often in pembrolizumab-containing perioperative regimens—remain standard for early TNBC and improve long-term outcomes.^[3]^ The relative taxane insensitivity in PD6 may reflect ABC transporter–mediated efflux, a recognized cause of reduced intracellular drug exposure.^[4]^ ZnPT (about 10 μmol/L) activates KCNQ (Kv7) channels, including KCNQ4, in patch-clamp assays,^[5]^ consistent with channel activation rather than guideline-based oncology use.^[6]^ Thus, one organoid’s taxane insensitivity does not define TNBC and highlights model variability and the need for orthogonal validation.

**Table 1:** Drug list and maximum concentrations

Drug name	Company	Cat.No	Target	Maximum Concentration (μmol/L)
Paclitaxel	TargetMol	T0968	Microtubule	10
Vinorebine	TargetMol	T0190	Microtubule	10
Docetaxel	TargetMol	T1034	Microtubule	10
Tamoxifen	TargetMol	T6906	Estrogen/progestogen Receptor	10
Epirubicin	TargetMol	T0125A	DNA/RNA synthesis	10
Doxorubicin hydrochloride	MedChemExpress	HY-15142	DNA topoisomerase I/II	10
Zinc Pyrithione	Absin	13463-41-7	KCNQ4	10
Cisplatin	TargetMol	T1564	DNA/RNA synthesis	100
Gemcitabine	TargetMol	T0251	DNA/RNA synthesis	100
Retigabine	Aladdin	R613161	KCNQ4	100

This study highlights dynamic HR expression in the MBC PDO and offers a lens on heterogeneity and treatment resistance. The observed Luminal A→TNBC-like shift during passaging underscores the limits of static biomarker assessment and suggests adaptive evolution—*via* epigenetic rewiring or clonal selection—once tumors are separated from the *in vivo* microenvironment. Because most MBCs are HR-positive, capturing HR dynamics in male models is important for biology and decision-making.^[7]^ Although breast cancer PDOs can expand long-term (> 20 passages) while retaining key histopathology,^[8]^ our complete HR loss by PD4 appears unusually early relative to drift more often linked to longer culture, supporting the hypothesis that microenvironmental deprivation and culture selection hasten HR independence and warrant validation in male cohorts.

Mechanistically, PD6 acquired alterations over the tumor in KMT2C, NBPF9, ANGPTL7, ARHGAP9, C9orf41, CAPS2, CUL7, DNER, FAM131C, and GINS1, while MUC4 exhibited non-overlapping alterations in the tumor (multi-hit) and in PD6 (a distinct missense), consistent with divergent evolution. These span epigenetic/ubiquitin regulation (*e.g*., KMT2C, CUL7) and signaling modulation (*e.g*., MUC4, DNER), but single-model data cannot establish causality for ER/ PR/AR loss or the phenotypic transition. Gene-set enrichment supported hormone and metabolic rewiring—Hallmark estrogen response (late) and cholesterol homeostasis (SREBP-related) —alongside cell-cycle programs (G2–M checkpoint). These signals suggest metabolic (cholesterol/SREBP) and hormonal axes are retuned with cell-cycle dysregulation; still, observations are correlative and require targeted functional studies. No direct evidence links NBPF9, ARHGAP9, CAPS2, or GINS1 to independent control of HR programs.

Building on these correlative signals, we highlight exploratory ZnPT-related mechanisms: prior reports implicate inhibition of proteasome-associated deubiquitinases and disruption of copper homeostasis with DLAT oligomerization (cuproptosis)^[9,10]^ and decreases in p-STAT3/CD44 and EGFR–PI3K–AKT have been described in TNBC models but were not assessed here.^[10]^ These hypothesis-generating leads align more with proteostasis/cuproptosis than with canonical HR signaling and do not establish a mechanism. Although active *in vitro*, ZnPT remains exploratory, and we also performed in silico database predictions (summarized in the Supplements 1 and 2). ZnPT is not guideline-recommended for TNBC; consistent with standards of care (*e.g*., pembrolizumab-containing taxane regimens for early TNBC), ZnPT should be treated as a preclinical probe pending validation in male cohorts and microenvironmentaware systems.

This work has limitations. As a single-case study, it cannot be generalized, and the receptor transition cannot be causally ascribed to specific variants or pathways. PD6 alterations (*e.g*., KMT2C, MUC4) and the apparent ZnPT activity may reflect clonal heterogeneity or culture-driven adaptation within a simplified *ex vivo* milieu lacking stromal, vascular, endocrine, and immune inputs; thus, anthracycline/platinum sensitivity and the ZnPT signal are context-dependent. Genetic drift during passaging may further drive HR loss and alter drug response *via* accrued changes in ubiquitin or cell-cycle regulators, affecting receptor programs and taxane sensitivity. To mitigate this, future work will build a small male-breast organoid cohort with longitudinal/ Lineage tracing, add stromal/ immune co-culture and parallel PDX validation, and apply multiomics plus targeted perturbations.

In summary, we document HR loss during *ex vivo* propagation of the MBC PDO, alongside genomic divergence and exploratory pharmacologic signals. The results support dynamic, context-aware classification in MBC research and nominate ZnPT only for preclinical investigation in TNBC-like states—pending rigorous validation.

## Supplementary Material

Supplementary Material Details
