# Supplementary material for: Dynamic loss of hormone receptors and reshaped drug sensitivity in male breast cancer organoids: A first case report and clinical implications
Source: J Transl Int Med. 2026 Apr 4;14(2):322–5. doi: 10.1515/jtim-2026-0032 (PMC13110457; doi:10.1515/jtim-2026-0032)

Supplementary materials

Supplement 1

Figure S1: Immunohistochemical (IHC) analysis was performed to detect the expression of AR, ER, and PR proteins in passage 4, 5, and 6 organoids.

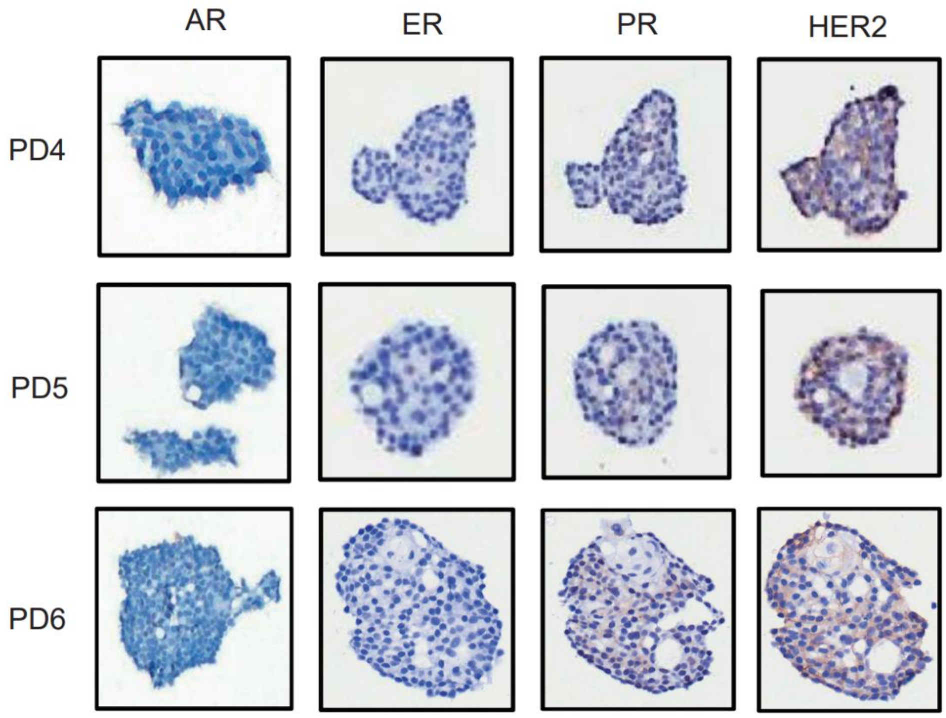

Figure S2: PD6-acquired variant categories relative to tumor; E. Enrichment of estrogen-response (late), SREBP/cholesterol, and cell-cycle (G2-M, mitosis) programs.

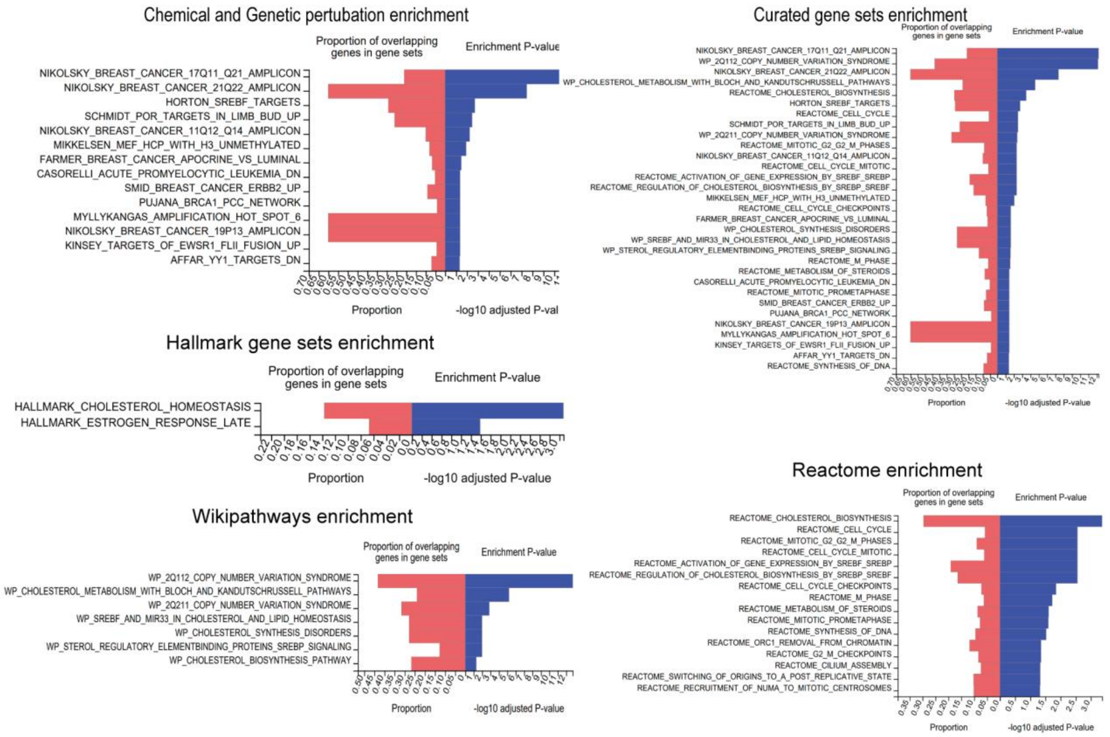

Figure S3

Table S1 & Figure S3: Compared with tumor tissues, genes mutated in PD6 organoids were subjected to correlation analysis in BRCA using GEPIA2, including AR, ER, and PR（Table1）. Figure A shows genes positively correlated with homologous recombination (HR), and Figure B shows genes negatively correlated with HR.

Gene	vs ESR1 (p/R)	vs AR(p/R)	vs PGR(p/R)	Note/Correlation
KMT2C	0/0.35	0/0.45	0/0.29	Significant/+
NBPF9	0/0.23	2.8e−08/0.15	0/0.23	Significant/+
C9orf41	0/0.35	0/0.38	0/0.23	Significant/+
CAPS2	7.1e−13/0.19	9.7e−14/0.2	0/0.26	Significant/+
CUL7	4.2e−10/0.17	2.4e−05/0.11	0.00067/0.092	Significant/+
GINS1	0.00076/0.091	0.0048/0.076	0.78/0.0075	Significant/+
MUC4	0.021/-0.062	0.0074/-0.072	0.39/-0.023	Significant/-
ANGPTL7	3.8e-10/-0.17	1.9e−06/-0.13	0.022/-0.062	Significant/-
ARHGAP9	1.4e−13/-0.2	3e−09/-0.16	0.0041/-0.077	Significant/-
DNER	0.0011/-0.088	3.9e−06/-0.12	0.32/-0.027	Significant/-
FAM131C	1.2e−09/-0.16	4.9e−21/-0.25	0.00019/-0.1	Significant/-

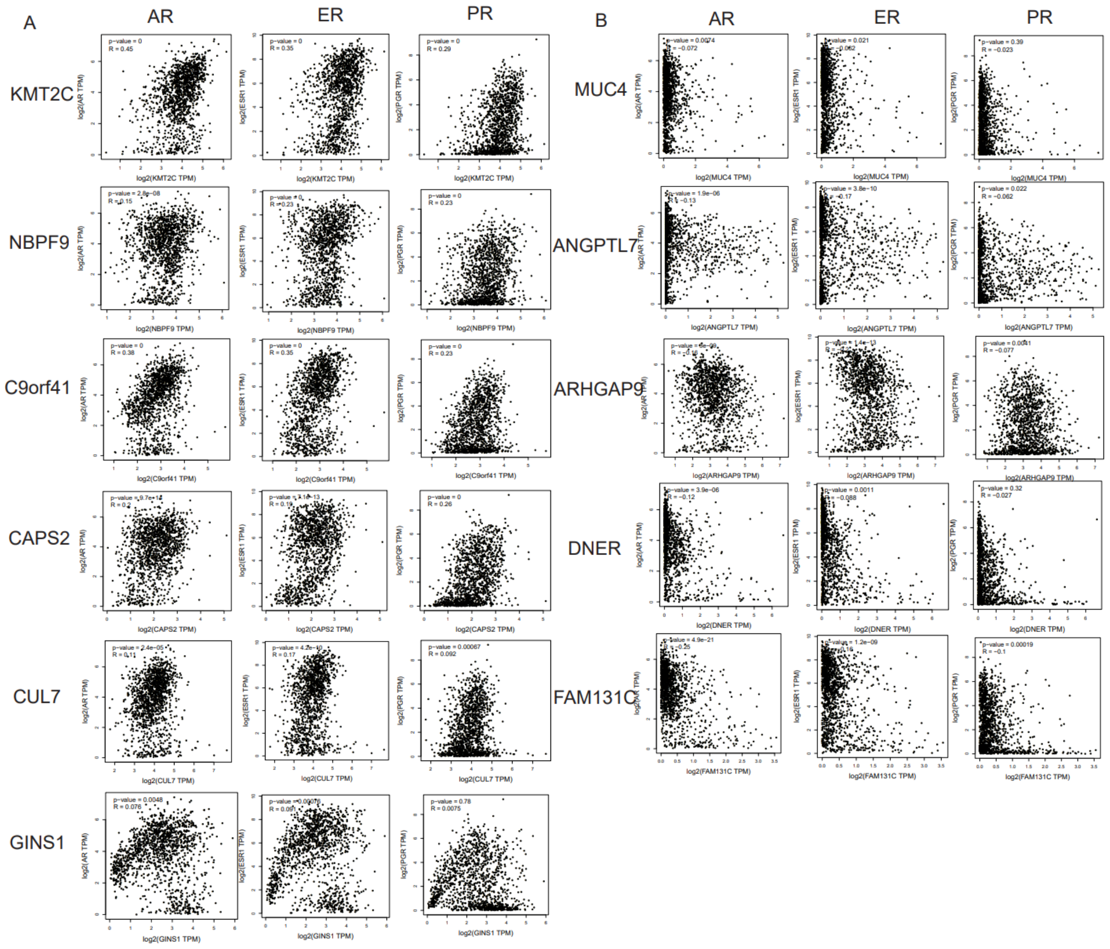

A
AR
ER
PR
KMT2C
NBPF9
C9orf41
CAPS2
CUL7
GINS1
B
AR
ER
PR
MUC4
ANGPTL7
ARHGAP9
DNER
FAM131C

Figure S4: Predict the sensitivity of three types of TNBC cell lines HLC13937, HDANB231, and HDANB468 to pyrithione-zinc (BRD:BRD-K16136380-001-01-7)ZnPT through the DepMap database. Drug sensitivity was quantified by AUC (Area Under the Curve) metrics from the PRISM Repurposing Secondary Screen. Data represent mean ± SEM of three independent experiments.Lower AUC values indicate higher drug sensitivity.

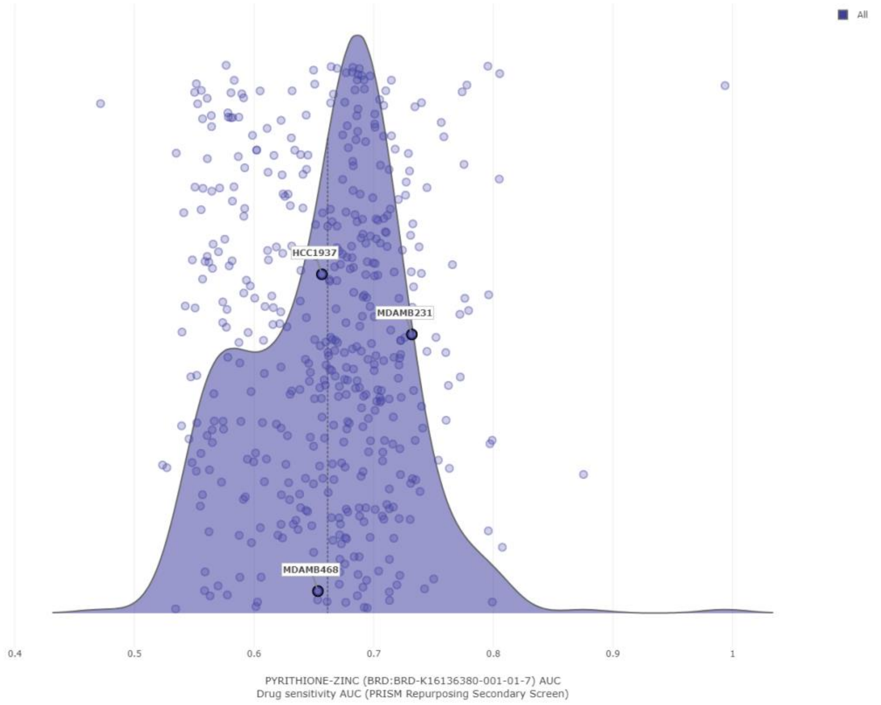

Supplement: Supplementary file 1 — Supplementary Material Details [file jtim-2026-0032_sm.zip › 14 JTIM-D-25-00223 SI 1.pdf]
