# Supplementary material for: Dynamic loss of hormone receptors and reshaped drug sensitivity in male breast cancer organoids: A first case report and clinical implications
Source: J Transl Int Med. 2026 Apr 4;14(2):322–5. doi: 10.1515/jtim-2026-0032 (PMC13110457; doi:10.1515/jtim-2026-0032)

Supplementary Materials

Supplement 2

We further conducted mechanistic investigations at the level of single nucleotide polymorphisms (SNPs) from PD6 sequencing, encompassing eQTL-associated genes, enhancers, promoters, and the genes regulated by SNPs associated with HI-C and ChIP.

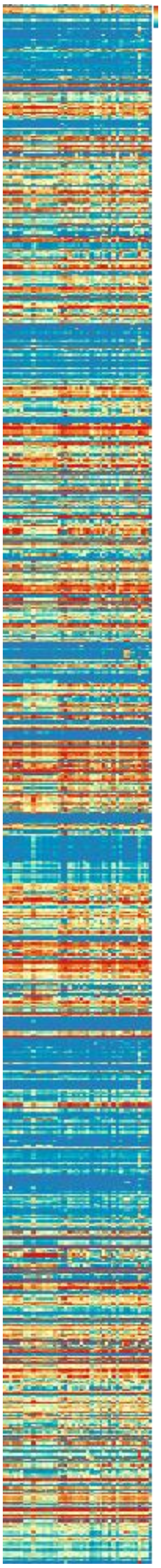

Supplement: Supplementary file 1 — Supplementary Material Details [file jtim-2026-0032_sm.zip › 14 JTIM-D-25-00223 SI 2.pdf]
